# Is blue dye still required during sentinel lymph node biopsy for breast cancer?

**DOI:** 10.3332/ecancer.2016.674

**Published:** 2016-09-19

**Authors:** Mirjam CL Peek, Tibor Kovacs, Rose Baker, Hisham Hamed, Ash Kothari, Michael Douek

**Affiliations:** 1 Division of Cancer Studies, King’s College London, Guy’s Hospital Campus, Great Maze Pond, London SE1 9RT, UK; 2 Guy’s and St. Thomas’ NHS Foundation Trust, Great Maze Pond, London SE1 9RT, UK; 3 School of Business, 612, Maxwell Bldg, University of Salford, Salford M5 4WT, UK

**Keywords:** breast cancer, sentinel lymph node biopsy, radioisotope, patent blue

## Abstract

**Background:**

In early breast cancer, the optimal technique for sentinel lymph node biopsy (SLNB) is the combined technique (radioisotope and Patent Blue V) which achieves high identification rates. Despite this, many centres have decided to stop using blue dye due to blue-dye-related complications (tattoo, anaphylaxis). We evaluated the SLNB identification rate using the combined technique with and without Patent Blue V and the blue-dye-related complication rates.

**Methods:**

Clinical and histological data were analysed on patients undergoing SLNB between March 2014 and April 2015. SLNB was performed following standard hospital protocols using the combined technique.

**Results:**

A total of 208 patients underwent SLNB and 160 patients (342 nodes) with complete operation notes were available for final analysis. The identification rate with the combined technique was 98.8% (*n* = 158/160), with blue dye alone 92.5% (*n* = 148/160) and with radioisotope alone 97.5% (*n* = 156/160). A total of 76.9% (263/342) of nodes were radioactive and blue, 15.5% (53/342) only radioactive and 2.3% (8/342) only blue, 5.3% (18/342) were neither radioactive nor blue. No anaphylactic reactions were reported and blue skin staining was reported in six (3.8%) patients.

**Conclusion:**

The combined technique should continue be the preferred technique for SLNB and should be standardised. Radioisotope alone (but not blue dye alone) has comparable sentinel node identification rates in experienced hands. National guidelines are required to optimise operative documentation.

## Introduction

In early breast cancer, sentinel lymph node biopsy (SLNB) is the preferred method for axillary staging [1–2]. SLNB involves identification and excision of the first lymph node(s) that receive lymphatic drainage from the primary breast tumour [3]. SLNB spares patients with preoperative negative nodes a more invasive axillary clearance operation and possible side-effects such as lymphedema, seroma, numbness, wound infection, reduced shoulder movement, and chronic pain [3–4].

The combined technique using both radioactive TcM99m and blue dye is the current golden standard for SLNB [5–6]. Identification rates of 96–97% were achieved in the AMAROS [3] and ALMANAC trials [7]. It is well known that use of the combination of radioisotope and blue dye is significantly better than the two tracers in isolation [7–9].

Despite the recognised superiority of the dual technique over radioisotope alone, many centres have decided to stop using blue dye due to blue-dye-related complications (tattoo, anaphylaxis) or lack of licensing for blue dyes [9–13]. We evaluated the SLNB identification rate in experienced hands using the combined technique with and without Patent Blue V and the blue-dye-related complication rates.

## Methods

Clinical and histological data were evaluated on patients undergoing SLNB between March 2014 and April 2015. This study was approved by the Clinical Review Board (ref 5320).

### Inclusion criteria

All patients who underwent SLNB for newly diagnosed breast cancer were included. In the case of a bilateral SLNB, only the side with the highest grade, largest primary tumour size or most involved nodes was included to avoid confounding variables.

### Exclusion criteria

Patients were excluded if there was no procedure description available in operative or pathology notes, if only an injection of radioisotope or Patent Blue V was administered, or if the colour or radioactivity within nodes was not documented.

### SLNB procedure

All SLNB procedures were performed using radioisotope, Patent Blue V and a scrubbed consultant surgeon. Patients scheduled to undergo SLNB in the Breast Unit at Guy’s and St. Thomas’ NHS Foundation Trust were injected with radioisotope on the morning of surgery either at the Nuclear Medicine Department or in Day Surgery Unit. Prior to surgery, Patent Blue V was injected peri-areolarly on table followed by a massage of the injection site. During SLNB, the surgeon used a gamma probe to detect the radioisotope and visually identified blue nodes. Any nodes with a count of 10% or more of the highest node count were excised. Beyond four nodes, surgeons excised additional nodes only at their discretion and noted the background count on completion. Palpable nodes felt during the procedure were also removed. For every excised node, the gamma probe was used to determine the radioactivity of nodes *ex vivo*. Operation notes included the amount of Patent Blue V injected, amount of nodes excised and if these nodes were blue and/or radioactive. A successful SLNB was defined as a procedure in which at least one true sentinel node was identified to be either blue and/or radioactive.

### Histopathology

All excised SLN’s were sent for histopathological evaluation in accordance with national protocols. Nodes were reported as either normal or involved containing isolated tumour cells (ITC) (≤ 0.2 mm), micrometastases (> 0.2 and ≤ 2.0 mm) or macrometastases (> 2.0 mm). The size of the largest deposit was also reported by the pathologist.

### Statistical analysis

To statistically determine whether SLNB with Patent Blue V provides a non-inferior identification rate compared to the combined technique, a sample size calculation was performed. With an identification rate for the combined technique of *p* = 0.97, we were prepared to accept non-inferiority with a margin of δ = 0.05. The probability that the addition of radioactivity results in finding new nodes is *r*_0_ = *kδ* with *k* = 0.2. With 90% power and a 95% one-sided confidence level, *z*_α_ is the standard normal variate value corresponding to the test size, *z*_α_ = 1.6449 and *z*_β_ the variate corresponding to the test power, *z*_β_ = 1.2816. Using the following formula gives a sample size of *N* = 148 patients.

$$N=\frac{\mathrm{(\{}\delta (1-\delta {\mathrm{)\}}}^{1/2}Z_{\alpha }+\{r_{0}(1-r_{0}{\mathrm{)\}}}^{1/2}Z_{\beta }{)}^{2}}{(\delta -r_{0}{)}^{2}}$$

A non-inferiority test was performed to define whether the blue dye technique alone is non-inferior to the combined technique using δ = 0.05 and *p* the identification rate of the combined technique. The probability of detecting a node with blue dye or with radioisotope alone should not be lower than *p*-δ. [14–15]

To evaluate potential differences in sentinel node identification with different tumour size and volume of blue dye, we performed an independent *t*-test. For tumour grade, lymph node status, the presence of lymphovascular invasion, ER/HER2 status, and surgeon’s experience, we performed a crosstab and Fisher’s exact test. We repeated the analysis for sentinel node identification with the combined technique, radioisotope alone, and blue dye alone.

## Results

A total of 208 patients underwent SLNB between March 2014 and April 2015. In the 48 patients who were excluded, operation notes were incomplete in 38 patients (18.3%) and operation notes were missing in eight patients (3.8%). One patient (0.5%) was excluded because she only received Patent Blue V and one patient (0.5%) only received radioisotope. A total of 160 patients in which 342 nodes (range 1–6 nodes) removed were available for final analysis. Patient and tumour characteristics are tabulated in Table 1.

### SLNB procedure

About 78.8% (126/160) procedures were performed by specialised breast surgeons who have over 10 years of experience and 21.2% (34/160) were performed by fellows specialised in breast surgery.

In 80 cases (50.0%), SLNB’s were performed on the left and 80 cases (50.0%) on the right. There were 12 cases (7.5%) of bilateral SLNB. SLNB was performed in combination with a mastectomy in 47 cases (29.4%), wide local excision (WLE) in 89 cases (55.7%), and re-excision in five cases (3.1%). In 19 cases (11.9%), an SLNB alone was performed.

### Volume of Patent Blue V dye injected

In 24 cases (15.0%), 1.0 ml of Patent Blue V dye was injected, 2.0 ml was injected in 70 cases (43.8%) and the volume of injected Patent Blue V dye was not documented in 66 cases (41.2%). All 1.0 ml injections were not diluted apart from one which was diluted with 2.0 ml of saline. About 40% (28/70) of 2.0 ml injections were not diluted and 60% (42/70) were diluted with 3.0 ml of saline.

### Identification rates

The sentinel node identification rate with the combined technique was 98.8% (*n* = 158/160), with blue dye alone 92.5% (*n* = 148/160) and with radioisotope alone 97.5% (*n* = 156/160) (Table 2a). In two patients (1.3%), the SLNB procedure failed to identify a sentinel node and an axillary node clearance was undertaken. The probability of detecting nine nodes or fewer (158–148) is 0.82 which does not meet the δ = 0.05, and therefore, the hypothesis of inferiority cannot be rejected for blue dye alone.

The extra number of nodes detected by radioisotope was 6.3% (CI = 95% (0.03, 0.11)) using the normal approximation of Agresti and Coull [16]. The combined technique therefore has a probability of 98.9% of identifying a node (CI = 95% (0.955, 0.995)), making it consistent with the results of the ALMANAC and AMAROS trials [16].

An independent *t*-test found no statistically significant difference in sentinel node identification between patients with different tumour size and volume of blue dye injected. For tumour grade, lymph node status, the presence of lymphovascular invasion, ER/HER2 status and surgeon’s experience, Fisher’s exact test found no statistically significant differences with sentinel node identification.

### Excised nodes

In 158 patients who underwent successful SLNB, a total of 333 sentinel lymph nodes were excised, a mean of 2.1 nodes per patient. One node was excised in 47 patients, two nodes in 68 patients, three nodes in 25 patients, four nodes in 16 patients, five node in one patient and six nodes in one patient. These nodes were both radioactive and blue in 263 nodes (79.0%), only radioactive in 53 nodes (15.9%), only blue in eight nodes (2.4%) and neither radioactive nor blue in nine nodes (2.7%) (Table 2b).

The patients with sentinel lymph nodes not detected by blue dye were injected with 1 ml of blue dye (9 patients, 11 nodes), 2 ml (20 patients, 30 nodes), or the amount was not mentioned (7 patients, 12 nodes). Patients with nodes not detected by either blue dye or radioisotope were injected with 1ml of blue dye (1 patient, 1 node), 2 ml (3 patients, 3 nodes) or this was not mentioned (4 patients, 5 nodes).

The two patients with a failed SLNB had nine nodes excised after injection of 2 ml blue dye (1 patient, 4 nodes) or this was not mentioned (1 patient, 5 nodes).

### Histopathology

On histopathological examination, 35 patients (16.8%) had involved lymph nodes, 15 patients (9.4%) had micrometastases and 20 patients (12.5%) had macrometastases (Table 2a). In these 35 patients with involved nodes, a total of 45 involved nodes were excised, two nodes (0.6%) with ITC, 18 nodes (5.3%) with micro-metastases and 25 nodes (7.3%) with macrometastases (Table 3a). A further nine patients (5.6%) had lymph nodes that contained nine (2.6%) ITCs.

Of 18 nodes with micrometastasis, 15 (83.3%) were both radioactive and blue and three (16.7%) were only radioactive. Of 25 nodes with macro-metastases, 18 nodes (72.0%) were both radioactive and blue, six nodes (24.0%) only radioactive and one node (4.0%) only blue (Table 3b). Therefore, the false-negative rate for blue dye was 21.4% (9/42 involved nodes) and for radioisotope 2.9% (1/34 involved nodes).

### Complication rate

Post-operative complications were seroma (*n* = 33), haematoma (*n* = 6), breast oedema (*n* = 2), infection (*n* = 2), erythema (*n* = 1), reduced shoulder movement (*n* = 1), pain (*n* = 1), a candida infection (*n* = 1) and an undocumented complication (*n* = 1).

Blue staining of the skin was reported in 3.8% (*n* = 6/160) of patients which successfully underwent SLNB and no anaphylactic reactions related to blue dye were reported.

## Discussion

The sentinel node identification rate of the combined technique was 98.8% in our study, in line with the identification rates reported in the most recent ALMANAC and AMAROS trials [3, 7]. The identification rates for blue dye and radioisotope alone were 93.1% and 97.5%, respectively. Radioisotope alone does not meet the a priori non-inferiority criteria and is thus non-inferior to the combined technique. However, in a few patients, sentinel nodes are still identified with blue dye alone, suggesting that the use of two different dyes increases sentinel node identification. The use of an additional tracer reduces the false-negative rate of sentinel node biopsy [10]. However, it might be possible to obtain the same SLNB false-negative rate by administering two radioisotope injections (an additional injection instead of blue dye) rather than one [17]. The combined technique should therefore remain the standard of care at present. No blue-dye-related serious adverse events were described, and six cases of blue dye staining were described, suggesting the blue dye staining is under-reported as it is often asymptomatic.

Variation was observed in the amount of blue dye administered. Furthermore, lack of adequate operative documentation during surgery was observed in 38 patients (18.3%). Documentation of retrieval of blue or radioactive nodes confirms if the SLNB procedure was successfully completed. There are currently no national (or international) guidelines describing the minimal data set required for the documentation of SLNB procedures. SLNB carries a 6–10% false-negative rate when performed using the standard combined technique, it is likely to be higher if the combined technique is not used with consequently lower identification rates. In this study, the false negative rates for blue dye and radioisotope were 21.4% and 2.9%, respectively. However, no axillary node clearance was performed, to be able to obtain the correct false-negative rate for both techniques. In order to be able to audit SLNB identification rates, it is essential to know the number of nodes removed, their colour and radioactivity count. Guidelines are needed to ensure standardised administration of blue dye and adequate documentation of these procedures, including the type, amount, and dilution of tracers used.

Blue dye causes a tattoo at the site of injection and carries a 0.6% risk of anaphylaxis. Complications due to blue dye may be underreported due to inadequate documentation. In this study, a blue tattoo was only reported in 3.8% (six patients) suggesting this is significantly under-reported as it is often asymptomatic. Furthermore, the blue staining was only reported up to three weeks after SLNB, without documentation of resolution. With the increased number of patients suitable for SLNB, it is important to reduce the risk of blue-dye-related complications. In order to understand the impact of the issue, correct documentation is necessary. Therefore, there should be greater emphasis on standardisation of surgical techniques.

With respect to nodal involvement the combined technique should be used since if only the radioisotope or blue dye techniques are used alone, involved nodes would have been missed (Table 3). The limitation of this study are the wide ranges of blue dye injection volumes and this could potentially impact on the identification rate. However, no statistically significant differences were found between sentinel node identification and volume of blue dye used and other factors, such as tumour size, tumour grade, lymph node status, the presence of lymphovascular invasion, ER/HER2 status, and surgeon’s experience. This is most likely due to the very small number of patients with no blue or radioactive nodes identified as the blue dye, radioisotope and the combined techniques have high identification rates.

In the search for new SLNB techniques, it will be difficult to find a technique with an identification rate superior to that of the combined technique in view of its very high identification rate. However, if we can develop a single agent or device that can accomplish the same level of accuracy and rule out the use of radioisotopes, it is likely that the combined technique will be replaced. Currently, several new techniques are under investigation, including the magnetic technique, using superparamagnetic nanoparticles and a hand-held magnetometer, showing an identification rate between 94.4% and 98.5% [12, 13, 18, 19] and a fluorescent technique, using indocyanine green and fluorescent cameras showing an identification rate between 73.8% and 100% [19–26]. These techniques are promising, but need further clinical evaluation. A randomised controlled trial comparing these outcomes against the combined technique would benefit these techniques. However, any randomised controlled trial will compare the new technique against the standard and it is important that the combined technique is used in a standardised way.

## Conclusion

The combined technique should continue to be the preferred technique for SLNB and should be standardised. Radioisotope alone (but not blue dye alone) has comparable sentinel node identification rates in experienced hands. National guidelines are required to optimise operative documentation.

## List of abbreviations

ALND: Axillary lymph node dissection
SLNB: Sentinel lymph node biopsy
WLE: Wide local excision
ITC: Isolated tumour cells

## Conflict of interest

“The author(s) declare that they have no conflict of interest.”

## Figures and Tables

**Table 1.** Patient and tumour characteristics.

Variable		Number of patients (%)
Type of surgery	Wide local excision Mastectomy Re-excision Sentinel node only	89 (55.7%) 47 (29.4%) 5 (3.1%) 19 (11.9%)
Tumour size	Mean ± SD (range)	18.9 ± 13.0 mm (0–75 mm)
Blue dye injected	1.0 ml 2.0 ml NR	24 (15.0%) 70 (43.8%) 66 (41.2%)
Tumour grade	1 2 3 NR	14.4% (*n* = 23) 41.9% (*n* = 67) 36.3% (*n* = 58) 7.5% (*n* = 12)
ER status	+ - NR	78.8% (*n* = 126) 18.8% (*n* = 30) 2.5% (*n* = 4)
HER 2 status	+ - NR	14.4% (*n* = 23) 78.8% (*n* = 126) 6.9% (*n* = 11)
Presence of lymphovascular invasion	+ - NR	23.1% (*n* = 37) 68.1% (*n* = 109) 8.8% (*n* = 14)

**Table 2.** Identification rate (a) per patient and (b) per node.

(a)		Blue dye *n* (%)		Total *n* (%)
		Yes	No	
Radioactive *n* (%)	Yes	146 (91.3%)	10 (6.3%)	**156 (97.5%)**
	No	2 (1.3%)	2 (1.3%)	4 (2.5%)
Total *n* (%)		**148 (92.5%)**	12 (7.5%)	160 (100%)

(b)		Blue dye *n* (%)		Total *n* (%)
		Yes	No	
Radioactive *n* (%)	Yes	263 (79.0%)	53 (15.9%)	**316 (94.9%)**
	No	8 (2.4%)	9 (2.7%)	17 (5.1%)
Total *n* (%)		**271 (81.4%)**	62 (18.6%)	333 (100%)

**Table 3.** Table 3. Histopathological outcome (a) per patient and per sentinel lymph node and (b) sentinel lymph node identification of involved nodes using radiosiotope or Patent Blue V.

(a)*	Per patient *n* (%)	Per sentinel lymph node *n* (%)
Normal	116 (72.5%)	288 (84.2%)
ITC	9 (5.6%)	11 (3.2%)
Micro	15 (9.4%)	18 (5.3%)
Macro	20 (12.5%)	25 (7.3%)

* ITC: isolated tumour cells, micro: micro-metastases, macro: macro-metastases.

**Table:** 

(b)*	Radioactive and blue *n* (%)	Radioactive alone *n* (%)	Blue alone *n* (%)	None *n* (%)
ITC	11 (100%)	0	0	0
Micro	15 (83.3%)	3 (16.7%)	0	0
Macro	18 (72.0%)	6 (24.0%)	1 (4.0%)	0

* ITC: isolated tumour cells, micro: micrometastases, macro: macrometastases.
